# Individuality Through Ecology: Rethinking the Evolution of Complex Life From an Externalist Perspective

**DOI:** 10.1002/ece3.70661

**Published:** 2024-12-06

**Authors:** Pierrick Bourrat, Peter Takacs, Guilhem Doulcier, Matthew C. Nitschke, Andrew J. Black, Katrin Hammerschmidt, Paul B. Rainey

**Affiliations:** ^1^ Department of Philosophy Macquarie University North Ryde New South Wales Australia; ^2^ Department of Philosophy & Charles Perkins Center University of Sydney Sydney New South Wales Australia; ^3^ Global Ecology Laboratory Partuyarta Ngadluku Wardli Kuu, College of Science and Engineering Flinders University Adelaide South Australia Australia; ^4^ School of Computer and Mathematical Sciences University of Adelaide Adelaide South Australia Australia; ^5^ Institute of General Microbiology Kiel University Kiel Germany; ^6^ Department of Microbial Population Biology Max Planck Institute for Evolutionary Biology Plön Germany; ^7^ Laboratoire Biophysique et Évolution, CBI, ESPCI Paris Université PSL, CNRS Paris France

## Abstract

The evolution of complex life forms, exemplified by multicellular organisms, can be traced through a series of evolutionary transitions in individuality, beginning with the origin of life, followed by the emergence of the eukaryotic cell, and, among other transitions, culminating in the shift from unicellularity to multicellularity. Several attempts have been made to explain the origins of such transitions, many of which have been internalist (i.e., based largely on internal properties of ancestral entities). Here, we show how externalist perspectives can shed new light on questions pertaining to evolutionary transitions in individuality. We do this by presenting the ecological scaffolding framework in which properties of complex life forms arise from an external scaffold. Ultimately, we anticipate that progress will come from recognition of the importance of both the internalist and externalist modes of explanation. We illustrate this by considering an extension of the ecological scaffolding model in which cells modify the environment that later becomes the scaffold giving rise to multicellular individuality.

## Graphical abstract

The evolution of complex life forms involves key evolutionary transitions in individuality, often explained through internal properties of organisms. This paper introduces an ecological scaffolding framework, emphasizing how external environmental factors contribute to these transitions, suggesting that progress will come from integrating both internalist and externalist perspectives.

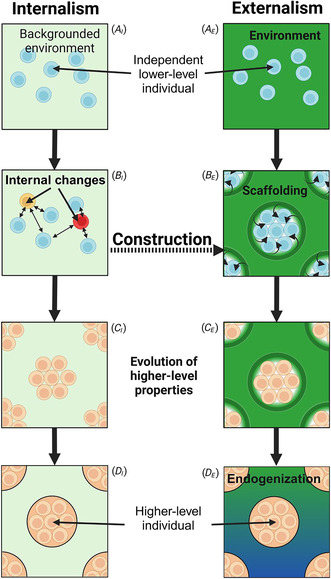

## 1 Introduction

Explanations for any biological construct can be internalist, externalist, or a combination of both (see Box 1 for further elaboration and Box 2). For example, consider a group of 100 cells that are packed in such a way as to produce a spherical structure. To explain the spherical organization, an internalist would likely resort to explanations invoking developmental mechanisms that are (internal) properties of the cells themselves. An externalist might argue that the spherical arrangement reflects the involvement of an external spherical structure into which the cells were packed.

BOX 1 **Glossary.**

**Collective**: Higher‐level entity constituted by lower‐level ones (e.g., a group of cells and an eukaryotic cell). Some collectives are Darwinian individuals.

**Darwinian individual**: Biological forms (e.g., unicellular organisms and multicellular organisms) endowed with the three Darwinian properties of discreteness, reproduction, and heredity (see Godfrey‐Smith 2009).

**Darwinian properties**: The properties exhibited by individuals at one level of organization that permit participation in the process of evolution by natural selection. We distinguish three properties that correspond to the three conditions for evolution by natural selection: *discreteness* underlying the condition of variation, *reproduction* underlying the condition of differential fitness, and *heredity* underlying the condition of heritability (see Godfrey‐Smith 2009; Black, Bourrat, and Rainey 2020; Bourrat 2014, 2022a).

**Ecological scaffold**: An environmental structure that enables individuals at one level of organization to realize Darwinian properties at a higher level of organization. In the example presented by Black, Bourrat, and Rainey (2020), the environmental scaffold is represented by bounded patches on which independent cells live and periodically disperse. A bounded patch containing a group of cells is effectively a discrete group that can reproduce (via a dispersal phase) with some heredity.

**Endogenization**: An evolutionary process during which higher‐level collectives gain the capacity to persist even in the absence of an ecological scaffold. They do so by developing properties that make the ecological scaffold unnecessary for their maintenance. In the case of the evolution of multicellularity, the evolution of an extracellular matrix maintaining the cells together in the absence of a scaffold represents an example of endogenization (see Black, Bourrat, and Rainey 2020; Bourrat 2022a; Doulcier et al. 2024).

**Evolutionary equilibrium**: A situation where a population has reached a state in which it does not evolve anymore (e.g., when mutations and selection are balanced; see Nowak 2006).

**Evolution by natural selection**: Process by which a population of individuals evolves under the action of natural selection. A common “recipe” for presenting this process involves three ingredients (more accurately, conditions): variation, differences in fitness, and heritability (see Bourrat 2014; Lewontin 1970; Godfrey‐Smith 2007; Bourrat 2015). This recipe is sometimes codified in the expression “heritable variation in fitness.”

**Evolutionary transition in individuality**: Phenomena that occur repeatedly in the tree of life where individuals at one level of organization interact and form a new level of individuality. Evolutionary transitions in individuality are also known as “major evolutionary transitions” and “major transitions” (although see Herron 2021 for a discussion of the distinction between those terms). Examples of evolutionary transitions in individuality are the transitions from cells to multicellularity or the transition to ancestral eukaroytic cells (see Black, Bourrat, and Rainey 2020; Michod 2005; Bourrat et al. 2022; Bourke 2011; Maynard Smith and Szathmáry 1995; Michod and Roze 1999; Buss 1987; Boomsma and Boomsma 2022; Bourrat 2021a; Bourrat 2019; Clarke 2014; Griesemer 2000).

**Fitness**: The currency of adaptive evolution. While many definitions of fitness exist and the concept is routinely debated in the philosophical literature (see Beatty and Finsen 1989; Sober 2001; Doulcier, Takacs, and Bourrat 2021; Takacs and Bourrat 2021, 2022), for our purposes, it represents the long‐term reproductive output of an individual in a particular environment.

**Fitness landscape**: A metaphor for the relationship between a phenotype (or genotype) and long‐term reproductive output (see Nowak 2006). Assuming that the effect of mutation is small, the action of natural selection can be visualized as a population climbing a hill in the fitness landscape. Once it reaches a peak, the population is at evolutionary equilibrium. In many cases, the relationship between phenotypic variation and long‐term reproductive output involves a landscape with more than one peak (of differing heights), prompting questions about crossing fitness valleys from one local peak to the other (see Doulcier et al. 2024 and Figure 2, for uses of the metaphor).

**Fitness transfer**: A proposed mechanism for the evolution of a new level of individuality. In the case of the evolution of multicellularity, cells can be considered as transferring their fitness to the multicellular entities that progressively gain individuality (see Michod 2005; Bourrat et al. 2022).

**Free‐rider problem**: Occurs when an individual (“cheater”) benefits from a public good but does not pay for it. This can lead to a tragedy of the commons. In an evolutionary context, the currency for costs and benefits is fitness (for a discussion of this problem in the context of evolutionary transitions in individuality, see Bourke 2011).

**Heritability**: Population‐level measure of heredity for a particular trait. Several definitions exist (see Jacquard 1983; Bourrat 2022b; Falconer and Mackay 1996; Lynch and Walsh 1998). In the context of this paper, it represents the extent to which offspring resemble their parent(s) more than they resemble other (non‐parental) individuals in the population (see Lewontin 1970; Godfrey‐Smith 2007).

**Inclusive fitness**: Fitness of an organism that considers both its direct offspring and the offspring of other individuals that carry the same alleles. Inclusive fitness is often invoked to explain the evolution of altruism where one individual sacrifices a portion of its direct reproductive output, which benefits other individuals carrying the same alleles (for introductions to this concept, see Bourke 2011; Futuyma 2005).

**Internalist**/**externalist mode of explanation**: Mode of explanation in which the internal or external properties of objects producing the phenomenon to explain are regarded as the operative causes. More detailed explanations of the distinction are provided in Godfrey‐Smith (1996) and Box 2. Internalism and externalism *do not mean* that internal or external operative causes, respectively, are the only causes required to explain a particular phenomenon. Any biological construct requires an interaction between a biological entity (through its components) and an environment. However, a particular explanation can be made while the particular value of a variable is kept constant or within a range that produces no change for the phenomenon to explain.

**Kin selection**: A selection process in which the indirect reproductive component of inclusive fitness is not negligible. A classic situation in which kin selection has been invoked is the evolution of altruism (for introductions to this concept, see Bourke 2011; Futuyma 2005).

**Multispecies individual**: A Darwinian individual that is composed of lower‐level individuals belonging to different taxa living in symbiosis (Bourrat and Griffiths 2018; Skillings 2016; Bordenstein and Theis 2015; Suárez and Stencel 2020; Ereshefsky and Pedroso 2013). Lichens are a classic example. Ancestral eukaryotic cells represent another example. They are thought to be the result of a transition involving ancestral eubacterial and archaebacterial cells (see Bourke 2011).

**Niche construction**: An evolutionary process by which an organism modifies its environment, which can affect future evolutionary trajectories (see Odling‐Smee, Laland, and Feldman 2003). In the ecology literature, the term “ecosystem engineer” is used to refer to species that modify their environment (e.g., Jones, Lawton, and Shachak 1994). More technically, niche construction can be conceived of as eco‐evolutionary feedback.

**Operative cause in an explanation**: A variable or set of variables in a setting for which a change in the value of this variable through intervention (e.g., experimental manipulation) permits to explain a particular phenomenon in the setting. Typically, in cases where more than one cause in a causal chain are considered as making a difference for this phenomenon, the cause on the chain that is further from the phenomenon to be explained will be considered the operative one (assuming here that the closer one is fully explained by the further one) and assuming there are no feedbacks involved in the explanation (see entry “Niche construction”). More than one operative cause can exist to explain a phenomenon. However, in experimental settings, putative operative causes (e.g., environmental fluctuations) are often controlled for (i.e., set at a certain value) and are therefore not considered part of the explanation.

**Superorganism**: A biological individual that is composed of multicellular organisms of the same taxon. Classical examples are beehives and ant or termite colonies (see Bourke 2011; Boomsma and Boomsma 2022).

**Tragedy of the commons**: A situation that occurs in some cases of free riding, where a public good is overexploited, which consequently leads to the demise of the system underwriting the public good (Hardin 1968). In the context of the evolution of multicellularity, a structure such as an extracellular matrix can be regarded as a public good that requires some investment from cells that benefit from this mode of reproduction. However, in the short term, each cell has an interest in not expending resources to produce the public good and instead invests resources in producing more offspring. This can lead to the demise of the multicellular organism, as is seen in cases of cancer, where cancerous cells are often depicted as free riders.

BOX 2 **The explanatory distinction between internalism and externalism.**

The distinction between internalist and externalist modes of explanation goes back to the work of Peter Godfrey‐Smith. In his 1996 monograph *Complexity and the Function of Mind in Nature* (Godfrey‐Smith 1996), he shows how explanatory debates about particular phenomena in the life sciences, often between two factions, occur. In general, “internalists” emphasize the internal components of the system as the primary explanatory factor (what we call “operative cause”). In evolutionary biology, works on self‐organization (Kauffman and Kauffman 1993; Goodwin 1994) or in the organicist tradition (e.g., Keijzer and Arnellos 2017; Mossio, Saborido, and Moreno 2009; Arnellos and Moreno 2015) typically resort to internalist modes of explanation. In contrast, “externalists” emphasize external components (i.e., the environment). Darwinian explanations, in which the environment plays an important role in explaining the emergence of particular traits through selective forces, are typically externalist. The internalist/externalist distinction, while general, should nonetheless not be regarded as absolute but rather as *relative* to the respective explanations being contrasted. There are internal and external components for every biological construct—every phenotype is the result of interaction between a biological system and its environment. However, emphasis is typically placed on one or the other. For instance, some Darwinian explanations, though typically more reliant on the role of the (selective) environment, may nevertheless emphasize properties of the entities in a population as doing the explanatory work.

A clear illustration of the foregoing point is the debate between two camps of evolutionary biology that emphasize different explanatory resources to explain eusociality. The evolution of eusociality producing so‐called “superorganisms” is arguably one of the most recent ETIs (Bourke 2011; Boomsma and Boomsma 2022; West et al. 2015). Assuming a textbook presentation of kin selection and inclusive fitness theory (e.g., Futuyma 2005), the evolution of altruism (i.e., increase in its frequency in the population) occurs when the fitness paid by individuals to partake in altruistic behaviors is compensated by benefits gained by their relatives (weighted by their relatedness). This is formalized as Hamilton's rule (Hamilton 1964). All these quantities and, in particular, relatedness (which measures the bias that altruists have toward preferential interaction with other altruists) are conceived primarily (although not exclusively) as dependent on mechanisms that are internal to the focal population, such as kin recognition, green beard effects, assortative mating, or clonality of reproduction (Bourke 2014). The role of the environment is accordingly “backgrounded” or averaged out, leading to extrapolation problems when calculating cost–benefit terms that are highly nonadditive (Smith, Van Dyken, and Zee 2010). This mode of evolutionary explanation can be traced back to Fisher (1930), Williams (1966), and Dawkins (1976). Some kin selectionists have accordingly argued for the importance of inclusive fitness in explaining the evolution of eusociality (Bourke 2011; Boomsma and Boomsma 2022; Queller and Strassmann 1998). In contrast, another group of researchers has emphasized the role of group selection, especially group formation, as opposed to kin selection (Wilson and Hölldobler 2005; Wilson 2008; Crozier et al. 1996; Nowak, Tarnita, and Wilson 2010). These scholars argue that the formation of a eusocial group is primarily driven by the influence of ecology, and the evolution of relatedness and particular payoff structures is a *consequence* of environmental structure.

**FIGURE 1:**
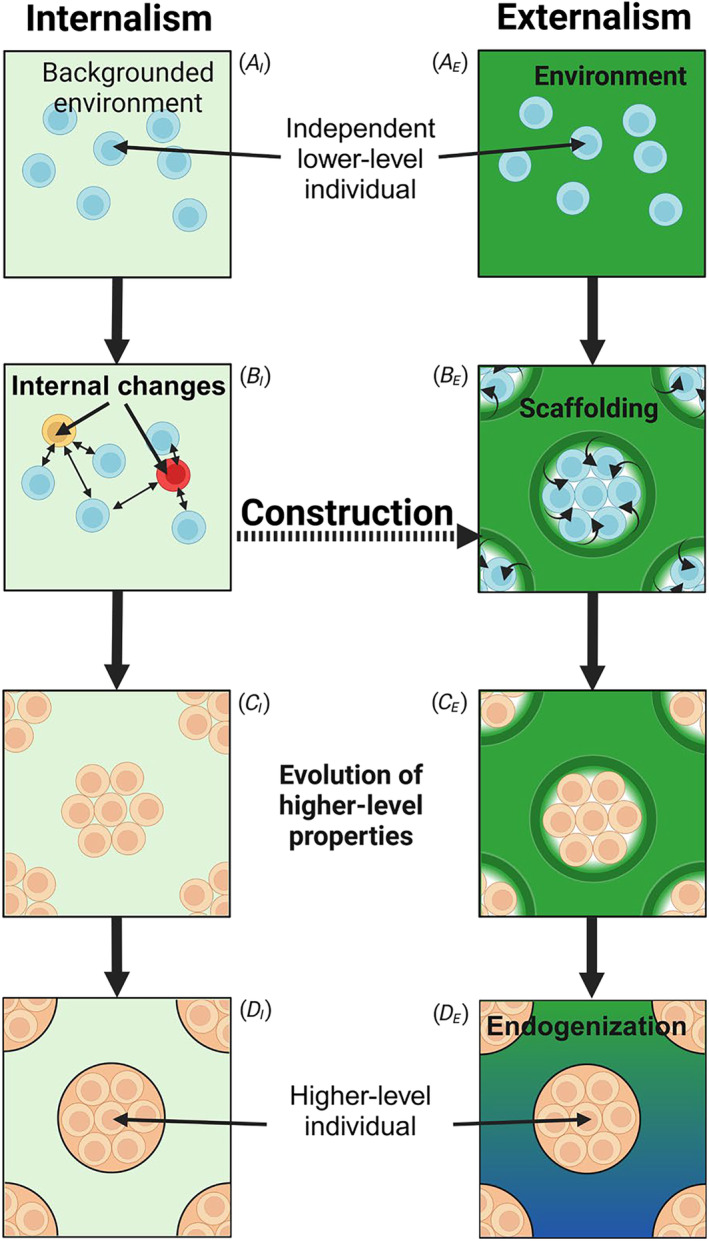
Internalist and externalist modes of explanation for the emergence of higher levels of individuality. *Internalism* starts by backgrounding the environment (*A*_*I*_) and considering whether some internal change in lower‐level entities (e.g., whether cells remain attached during division) (*B*_*I*_) will lead to the evolution of higher‐level properties (*C*_*I*_) that will ultimately lead to the evolution of higher‐level individuals, such as multicellular organisms (*D*_*I*_). *Externalism* starts by making the environment explicit (*A*_*E*_) and considering whether specific environmental structures (i.e., scaffolds) (*B*_*E*_) will lead to the evolution of higher‐level properties (*C*_*E*_) and ultimately to higher‐level individuals (*D*_*E*_). Additionally, externalism emphasizes the importance of the stability of higher‐level properties in environments other than the originating environment (*D*_*E*_). A bridge between internalism and externalism can be drawn via construction (*B*_*I*_) and (*B*_*E*_) by considering a situation where internal changes in lower‐level individuals lead to modifications of the environment that subsequently follow the externalist path.

Turning to evolutionary transitions in individuality (ETIs), exemplified by those from unicellularity to multicellularity (Grosberg and Strathmann 2007; Herron, Conlin, and Ratcliff 2022; Niklas and Newman 2016)—both internalist and externalist explanation are possible, however, causality is most commonly attributed to internal factors (see Figure 1 *A*_*I*_ to *D*_*I*_). Some emphasize mechanisms that concern the maintenance of cooperation and solutions to the free‐rider problem (Bourke 2011; Boomsma and Boomsma 2022). Other explanations emphasize the role of topological constraints or internal constraints in cell–cell interactions (Yanni et al. 2020; Keijzer and Arnellos 2017). Experimental studies have shown the importance of multicellular group formation via mutations that hinder cell separation after division (Ratcliff et al. 2012; Hammerschmidt et al. 2014; Rose et al. 2020), as well as revealed the importance of selection on developmental programs that determine life cycles replete with reproductive division of labor (Hammerschmidt et al. 2014; Rose et al. 2020).

In the case of multispecies individuals (see Bourrat and Griffiths 2018; Skillings 2016; Bordenstein and Theis 2015), explanations for why two or more independently reproducing individuals form a new larger self‐reproducing collective are often given in terms of benefit gained by each partner. While such explanations are perfectly sound, they tend to underplay the role of the environment in bringing together or maintaining collectives of lower‐level individuals. For example, in the case of lichens that are composed of at least two partners (a fungus paired with an algal or cyanobacterial species), neither partner can live independently in the environment where lichens are found (Kranner et al. 2005).

In more recent years, a school of thought has developed that emphasizes the role of the environment during ETIs under the label “ecological scaffolding.” As others have noted (see Veit 2021; Neto and Meynell 2023; Neto, Meynell, and Jones 2023), development of the ecological scaffolding framework has marked a shift in the kinds of explanations available for the evolution of multicellularity (and other ETIs). While commonly accepted internalist explanations recognize that the operative causes are internal to the individual entities or populations they constitute (Bourrat 2022a; Veit 2021; Neto and Meynell 2023; Neto, Meynell, and Jones 2023), ecological scaffolding provides an externalist approach where ecological factors have explanatory value (Black, Bourrat, and Rainey 2020; Bourrat 2022a, 2024; Doulcier et al. 2024, 2020; Nitschke et al. 2023; Jones et al. 2023; Griesemer and Shavit 2023; Libby and Ratcliff 2014; Libby et al. 2016) (see Box 2 and Figure 1). Ecological scaffolding builds upon developments in the field of experimental evolution (e.g., Ratcliff et al. 2012; Hammerschmidt et al. 2014; Rose et al. 2020; van Gestel and Tarnita 2017; Brunet et al. 2021; Kapsetaki and West 2019; Rainey et al. 2017) and philosophical work on cultural evolution, reproduction, and inheritance (Griesemer 2000, 2014; Caporael et al. 2013; Sterelny 2003; Wimsatt and Griesemer 2007).

Here, we focus on the formation of collectives during ETIs, with this step representing a necessary stage in the evolution of more complex forms of collective‐level individuality.

One of the main drivers motivating a switch in perspective from internal to external was the recognition of a central problem underpinning ETIs: namely, the origin of (Darwinian) properties sufficient to allow nascent collectives to participate in the process of evolution by natural selection—as units of selection in their own right (although see Michod 2005; Michod, Nedelcu, and Roze 2003 for a more internalist exception). Traditionally, the literature on ETIs has focused on the evolution of cooperation as a central problem, with solutions relying heavily on Hamilton's recognition of inclusive fitness (Hamilton 1964). This class of explanation involves an actor performing an altruistic behavior, a recipient, and a coefficient of relatedness (Bourke 2011; West et al. 2015) (see Box 2 and Discussion for more on this point). For example, cells in a multicellular organism must cooperate in order for the higher‐level entity to function (Bourke 2011; Buss 1987; West et al. 2015). Generally, environmental conditions, while in the background of the explanation, are not considered an operative cause in the initiation of an ETI, unless they promote cooperation between members of groups. However, cooperation alone is insufficient to produce collective‐level individuals. The externalist camp, in contrast, has focused on the evolution of Darwinian properties, seeing that cooperation and solutions to the free‐rider problem emerge as consequences of the group becoming a unit of selection.

Following Lewontin (1970) (see also Godfrey‐Smith 2007), evolution by natural selection at a given level of organization typically occurs when a population of biological objects satisfies three conditions: (1) phenotypic variation that leads to (2) differences in fitness that are (3) heritable. For these three conditions to be fulfilled, the objects forming a population must be endowed with the three following properties, which constitute what we refer to as “Darwinian properties” (see Box 1): discretization (thus making variation possible); reproduction (which permits differences in fitness); and heredity (underlying heritability). However, specifying exactly how these Darwinian properties emerge at a new level of organization is a nontrivial matter (Bourrat 2021a, 2021b; Okasha 2006).

In some cases, the emergence of the three Darwinian properties at the higher level may emerge by co‐option of lower‐level traits, as in cases where cells do not fully separate after division (Ratcliff et al. 2012). However, in other cases, such possibilities may not exist. Grappling with the latter and recognizing that natural selection at the collective level cannot be invoked to explain the emergence of Darwinian properties at the same level—doing so would assume that which requires explanation as the cause of its own evolution—led to development of the “ecological scaffolding” framework, which emphasizes the role of external factors in ETIs (Figure 1 *A*_*E*_ to *D*_*E*_). This conceptual framework, which has both experimental (Hammerschmidt et al. 2014; Rose et al. 2020; Rainey 1872) and theoretical (Black, Bourrat, and Rainey 2020; Doulcier et al. 2024, 2020) support, emphasizes that higher‐level Darwinian properties can be exogenously imposed by specific ecological conditions.

Thus, while internalist and externalist explanations are complementary and overlapping, they have, until the present time (and in part attributable to the historical development of ideas), tended to address different problems. The uninitiated reader should therefore be aware that when we contrast internalist and externalist explanations of ETIs, the two different perspectives typically do not provide contrasting explanations for the same problem. Given these differences in explanatory targets, we advocate the value of embracing externalist perspectives: not only do such perspectives allow for the resolution of problems commonly associated with internalist explanations but also one of the additional benefits is the opportunity to generate new hypotheses about the origins of life's hierarchical structure. We further contend that externalist perspectives can be extended and combined with internalist approaches, opening up new avenues for theory and experimentation.

## 2 Ecological Scaffolding and the Value of Externalism

Applied to the evolution of multicellularity, the ecological scaffolding framework proposes that ETIs can be initiated by specific environmental conditions that exogenously impose Darwinian properties of variation, reproduction, and heredity at higher levels of organization. A simple example (see Black, Bourrat, and Rainey 2020) involves an environment that is structured into bounded patches filled with resources on which cells grow. When resources are depleted, cells die. Periodically, some cells are dispersed as propagules, providing an opportunity for those cells to colonize unoccupied patches. The number of propagules that cells within a patch provide to the next generation depends on population size at the time of dispersal (which in turn depends on the rate of resource consumption relative to the timing of dispersal events). The presence of patchily distributed resources and a means of dispersal are sufficient for cells within patches to become unwitting participants in a selective process that takes place within, as well as between, patches (Black, Bourrat, and Rainey 2020). Primordial multicellular organisms exhibit variation in the growth rate of the cells that compose them. This variation leads some to produce more propagules at the time of reproduction. Finally, primordial multicellular organisms pass properties on to their offspring. Accordingly, natural selection can optimize traits of lower‐level individuals to prevent overexploitation of resources within patches, thereby facilitating the colonization of new patches (Black, Bourrat, and Rainey 2020).

Finding actual cases that correspond to this ecological scenario is inherently difficult, simply because a scaffold is a transient feature that disappears over time, an outcome of what we call “endogenization” (see Section 3 and Box 1). Notwithstanding this difficulty, an intuitive example would involve cells that live in tidal pools filled with resources from the sea. As the tides ebb and flow, pools are replenished with resources, and some cells disperse from one pool to another. Another example elaborated in Rainey et al. (2017), which inspired an experimental setting (see Hammerschmidt et al. 2014), involves a pond with reeds that extend from the water. Reeds provide a scaffold for bacteria capable of forming mats through the production of a polymer. Each mat can collapse due to external disturbance or internal factors (e.g., the bacteria not producing the polymer in sufficient amount to maintain structural integrity of the mat) and open a new niche for bacteria on neighboring reeds to colonize. A further example concerns obligate microbial pathogens and commensals, particularly those that go through restrictive bottlenecks at each infection. Here, hosts are patches of resources, which ensures discretization of microbial groups, and transmission is akin to reproduction (with heredity). Although it is not usual to consider obligate pathogens and commensals as multicellular, ecological scaffolding predicts that we should not be surprised to find evidence of a reproductive division of labor among certain microbial groups (see Black, Bourrat, and Rainey 2020 and references therein).

These simple settings, which approximate the model proposed by Black, Bourrat, and Rainey (2020), can drive the evolution of multicellularity (Hammerschmidt et al. 2014). Departing slightly from these settings, another example is the green algae *Ulva australis,* which thrives in intertidal zones. Synergistic interactions of multiple species of bacteria generate biofilms on the algal surface that provide resistance to invasion by other species and protection from stressful conditions (Burmølle et al. 2006). Following this example, a population of green algae in an intertidal zone could be regarded as consisting of patches with resources and multispecies biofilms as proto‐multicellular organisms. While the proposal to regard multispecies biofilms as Darwinian individuals may be contentious, biofilms exhibit Darwinian properties to some extent (Ereshefsky and Pedroso 2013). As such, they partly satisfy the conditions for being regarded as higher‐level proto‐individuals with ecological scaffolding as a possible mechanism promoting the evolution of clearer forms of higher‐level individuality.

Following the ecological scaffolding framework, scaffolding conditions cause collectives of lower‐level entities to experience selective conditions similar to those if they were higher‐level individuals, by means that are entirely exogenous to lower‐level entities. For this reason, this framework recognizes the possibility that conditions facilitating the evolution of higher‐level entities may be externally (exogenously) imposed. At its core, the distinguishing feature of the ecological scaffolding framework is the contrast it draws between a situation in which there is no ecologically imposed population structure and a situation in which the scaffold is present. Internal changes do occur along with the scaffolding, such as mutations that reduce the growth rate of the cells in the model of Black, Bourrat, and Rainey (2020). However, they are merely “follower” factors in the causal chain (and therefore explanation). In other words, internal changes are not operative causes in explaining the transition. This mode of explanation contrasts with alternative internalist models based on fitness transfer (Michod 2005), kin selection (Bourke 2011; Boomsma and Boomsma 2022), or incomplete separation during cell division (Ratcliff et al. 2012), where internal changes are “leading” factors or operative causes in the causal chain.

Note that factors such as selection for larger size leading to lower predation (see Kirk 1998; Herron et al. 2019) or faster sedimentation time (see Ratcliff et al. 2012) in internalist models are clearly external to cells and are involved in the production of boundaries and the evolution of higher‐level individuality. However, they follow internal changes to would‐be higher‐level individuals. For instance, the selective role of external factors can occur only if complete cell separation after division fails. Internal changes can thus be “singled out” as making a difference to whether a given transition is initiated and therefore be regarded as operative causes of the explanation.

While internalists and externalists tend to (de)emphasize distinct aspects of explanation, neither would deny that both environmental variation and changes in internal properties of biological entities have some explanatory role to play. Considering this recognition, one might well wonder about the value of switching from a primarily internalist to a more externalist mode of explanation for ETIs. Instead, one might reason that both external and internal changes should be taken into account and that one need not assume that the factors initiating a transition are either external or internal; a general model should account for the possibility that they might well be both.

We certainly agree and discuss one such scenario involving both types of factors in Section 4. However, it is important to acknowledge that scientific explanations are not what philosopher Peter Railton called “ideal explanatory texts”: that is, complete explanations for given phenomena (Railton 1981). Instead, they are inevitably incomplete and may present different aspects of biological reality. Further, it is crucial to note that, in a context where many of the explanations offered (mentioned above) have focused on the internal components of higher‐level entities, the proposal of an externalist perspective and the development of the ecological scaffolding model may provide solutions to longstanding problems or pose new questions and lead to further developments. Privileging internal over external causes for explanation appears to be a general feature of human cognition (see Cimpian and Salomon 2014 and references therein). Thus, the development of an externalist approach to ETIs is not only compatible with but also complementary to the internalist approach.

A primary motive for developing the ecological scaffolding framework was to provide a satisfactory solution to a shortcoming of more internalist explanations for the evolution of multicellularity and other ETIs: namely, that they take the existence of higher‐level individuals and Darwinian properties for granted when, in fact, it is the very existence of Darwinian higher‐level individuals that must be explained (Black, Bourrat, and Rainey 2020; Bourrat 2022a; Clarke 2014; Rainey et al. 2017). The emergence of higher‐level Darwinian properties from lower levels is a problem encountered when examining any ETI. We have already presented putative scenarios for the evolution of multicellularity in intertidal zones that would provide conditions for the potential emergence of Darwinian properties at the level of multicellular proto‐organisms. However, the problem is particularly salient for the emergence of the first biological systems. Prebiotic molecules are particularly simple, and inherent heritable variations in fitness are difficult to conceive of. Nevertheless, it becomes more plausible if assortment between molecules is imposed by the physical structure of the environment, as would be the case when following the ecological scaffolding framework. For example, a lattice of iron monosulfide precipitate in hydrothermal vents creates pores that can play the same role as cell walls (Martin and Russell 2003).

In developing the ecological scaffolding framework, there was never any intention to argue that it is the only solution to the problem of the origins of Darwinian properties. As was already mentioned, some more internalist explanations, such as those that involve incomplete cell separation due to a mutation, can likewise generate higher‐level variation, differences in fitness, and heritability without the existence of a scaffold and without presupposing the existence of collective‐level Darwinian properties. Rather, the main goal is to provide a novel solution to a problem that other approaches could not adequately address because their explanatory commitments diminished the importance or viability of any such externalistically inclined solution.

This last point also provides a partial answer to those who would argue that a purely internalist view is sufficient to account for any ETIs. We agree, as mentioned above, that any phenomenon can be explained by foregrounding or backgrounding particular factors. Ultimately, the distinction between the internalist and externalist mode of explanation represents a shift in *perspective*, similar to the shift in perspective advocated by Dawkins (1982) with the Necker cube analogy: to shift from an organism‐centered view of evolution to a gene‐centered one. Although a shift in perspective is not, in and of itself, a testable hypothesis, it can be useful to generate new hypotheses. For instance, a purely internalist approach might lead to building models or designing experiments in which the environment is considered constant. The ecological scaffolding model, because it starts from a more externalist perspective, demonstrates not only that the environment can be at least as important as internal factors—and therefore should not be neglected—but also that it can provide adequate explanations for phenomena otherwise difficult to explain. The next section illustrates this point.

## 3 Endogenization as a Puzzle for Evolutionary Transitions in Individuality

Historically, a major challenge to the evolution of higher‐level individuality is the so‐called “free‐rider” problem, which leads to the tragedy of the commons (Hardin 1968): how can a higher‐level individual evolve if cooperation between the lower‐level individuals composing it is susceptible to invasion by free riders who reap short‐term benefits and thus undermine the formation of any higher‐level entity? The internalist answer to this tragedy lies in the evolution of mechanisms internal to the higher‐level individual (conflict mediators) that prevent such an event (Michod, Nedelcu, and Roze 2003). Without a solution to the free‐rider problem, according to this view, no higher‐level individuality can evolve.

Seen through the lens of ecological scaffolding, the tragedy of the commons becomes secondary. The challenge instead lies in the observation that modern organisms exhibit the three Darwinian properties and that, perhaps unlike their ancestors, these properties are exhibited without the need for a scaffold. Based on this observation, the puzzle of the evolution of higher‐level individuality becomes the origin of scaffolding conditions that permit Darwinian properties to manifest at the higher level and endogenization of these properties if the scaffold is lifted, as emphasized in several experimental and theoretical publications within the ecological scaffolding framework (Black, Bourrat, and Rainey 2020; Hammerschmidt et al. 2014; Rose et al. 2020; Rainey et al. 2017; Doulcier, Hammerschmidt, and Bourrat 2020; Doulcier et al. 2024). This is tantamount to asking how ecologically scaffolded collective individuals can acquire the ability to escape the confines of the environment that gave rise to them and thereby persist under novel or different ecological conditions.

As has already been emphasized, these internalist and externalist modes of explanation—with the respective questions they raise—should not be seen as existing in opposition. Their interdependence becomes obvious as soon as it is recognized that the maintenance of cooperation (via mechanisms of conflict suppression) between lower‐level individuals within a higher‐level entity is necessary for successful endogenization. Similarly, while the emergence and maintenance of cooperation is important for ETIs, in and of itself, this does not provide a solution to the emergence of Darwinian properties, as is emphasized by the ecological scaffolding framework. Thus, in principle—although this is rarely done in practice—the two modes of explanation can be regarded as emphasizing different aspects of ETIs and, accordingly, providing different explanatory strategies.

**FIGURE 2:**
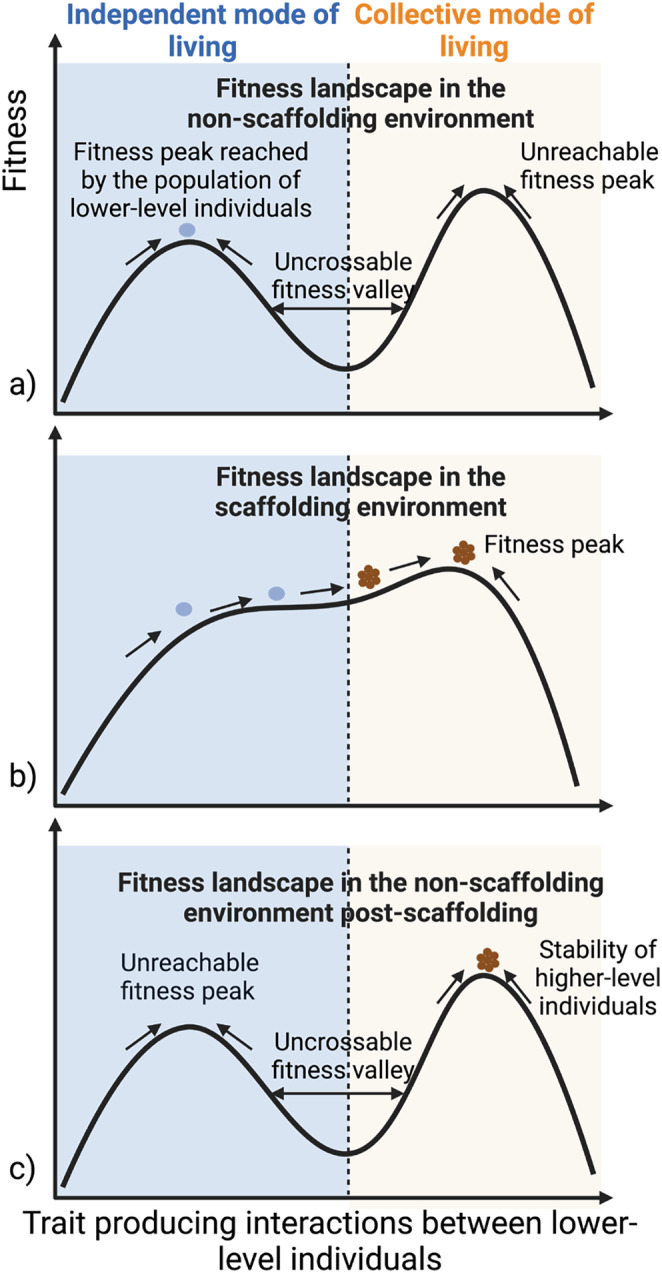
Conditions for endogenization during an evolutionary transition in individuality by ecological scaffolding, based on the model by Doulcier et al. (2024). (a) The fitness landscape contains two peaks separated by an uncrossable valley. A population of independent lower‐level individuals optimizes for traits favoring this mode of living. (b) Under the existence of an ecological scaffold, which might occur temporally or spatially, the fitness landscape is changed so that only one peak exists, favoring a collective mode of living: that is, higher‐level individuality. (c) After the removal of the scaffold, assuming the population has climbed sufficiently close to the peak favoring a collective mode of living, it cannot return to an independent mode of living due to the reappearance of the valley.

Delineating the environmental conditions required for endogenization and the emergence of individuality at higher levels provides a vivid illustration of how the ecological scaffolding framework foregrounds external factors for the stability of higher‐level entities at the expense of more internal ones. In addition to the existence of a non‐scaffolding environment and a scaffolding environment either over time or across space, endogenization requires the existence of at least two types of evolutionary equilibria in the non‐scaffolding environment. This could be conceived in different ways, such as a patch containing only two types of resources (note that this example is different from Doulcier et al. 2024). Assume that the first can be exploited by any independent cells on a patch, while the second can only be exploited if the cells act in a coordinated way (i.e., as a higher‐level individual). Further, it is assumed that exploiting the second type of resource provides a growth advantage with the constraint that switching from the first to the second type of resource involves a short‐term fitness disadvantage. Using the fitness landscape metaphor (see Figure 2a), each equilibrium represents a fitness peak—one favoring traits for lower‐level individuals with an independent mode of living, while the other promotes traits for lower‐level individuals with a collective mode of existence—separated by a valley that cannot be traversed (Doulcier et al. 2024).

Within this framework, one important obstacle to a collective mode of living is passage across fitness valleys. The (externalist) solution, starting from the ecological scaffolding scenario, is to assume that the environment experienced by cells may differ spatially and/or temporally, leading to local circumstances in which the first peak is effectively eliminated (or attenuated) (see Figure 2a,b). Under these circumstances, climbing the second peak or becoming extinct are the only possible endpoints. Assuming the former, a collective mode of life is likely to evolve within patches. Once a multicellular mode of living has evolved, there is no going back. The fitness peak for the second type of resource, once exploited, is higher than that of the first type. From then on, cells behave as parts of a higher‐level individual, even if the conditions return to their previous state and the two peaks are restored.

By emphasizing that the evolution of higher‐level individuality has only truly occurred if a return to an independent mode of existence for cells is hindered upon the removal of the scaffolding conditions, the ecological scaffolding framework motivates a number of new questions that a focus on internal properties alone would not raise. For instance, it encourages research on the ecological conditions that might lead to or prevent a return to an independent way of life for cells. This can be accomplished without explicit reference to the tragedy of the commons. Again, this is not to say that the tragedy of the commons is not a constraint during some ETIs. It is simply to point out that the externalist explanation does not emphasize the challenge posed by the tragedy of the commons.

Additionally, the ecological scaffolding scenario highlights the possibility of viewing the evolution of higher‐level individuality not only as a temporal sequence of events but also as a spatially distributed process, whereby some small regions of the total environment (“Goldilock zones”) can act as initiators of ETIs (Doulcier et al. 2024). Again, this type of explanatory resource is not of particular interest for internalist explanations.

## 4 Combining Changes in Internal and External Properties

We opened this piece by presenting two basic modes of explanation that could be invoked to account for ETIs: internalist and externalist. However, both internal and external factors should be considered equally important initiators of ETIs. This is evident, for example, in the modern understanding of kin selection, as discussed above and in Box 2, where “costs” and “benefits” are statistically interpreted (Bourke 2011; Birch 2014), rather than being defined solely by the internal properties of individuals. There are many causal relationships involving both internal and external relationships that can give rise to the same statistical relationship. For instance, effects external to populations (e.g., meta‐population structure due to resource patchiness) can be included in the computation of relatedness (e.g., see Jansen and Vitalis 2007). The line between internalist and externalist explanation consequently blurs as “costs” and “benefits” become properties of the entire population in its environment at a given point in time. The notion of inclusive fitness has been similarly generalized in adaptive dynamics, which fully accounts for ecological factors when making predictions (Ferriere and Michod 2011). Thus, for the most sophisticated evolutionary tools, the internalism/externalism distinction might not always be relevant. However, it nevertheless remains a good starting point for assessing which factors might be emphasized in a particular evolutionary explanation. Moreover, most formal tools arguably have their origin in either more externalist or internalist schools of thought (see Box 2 for an example).

A second way to challenge the strict division between internalist and externalist modes of explanation is by extending the “pure” ecological scaffolding scenario to include internal changes whose subsequent environmental effects become a scaffold (i.e., *constructive* factors). In the pure ecological scaffolding scenario, only causal arrows from the environment to lower‐level individuals explain change from the independent to a collective mode of living (Figure 1). In its constructionist incarnation (Figure 1, path from *B*_*I*_ to *B*_*E*_), ecological scaffolding works similarly, but the origin of the scaffold is assumed to be internal since the lower‐level individuals “construct” or, more neutrally, “modify” their environment, which later becomes the scaffold, following the literature on niche construction (Odling‐Smee, Laland, and Feldman 2003).

According to this scenario, the discretization of the environment into patches containing resources is the result of the activities of cells. For instance, we could assume that lower‐level individuals produce waste that accumulates at the edge of the resources as they feed. As the waste accumulates at the edge of the resources, cells may become trapped and lose their ability to move freely and feed elsewhere, as is the case with biofilms (Lyons and Kolter 2015). This phenomenon could create patches with increasingly more defined boundaries and lead patches of proto‐multicellular organisms to evolve traits at that level. Those traits might allow the exploitation of secondary resources that cannot be exploited by independent cells and therefore provide a long‐term advantage, as in the purely externalist ecological scaffolding scenario. Future formal models will elaborate on this idea.

Further insights can be gained by following the ecological scaffolding scenario via the constructionist path. When cells “construct” their environment, the constructed environment is unlikely to remain static. Shells provide an example of an environment that is not static but is nevertheless tightly coupled to the organism. One controversial hypothesis is that carbonated shells arose as a by‐product of the detoxification of intracellular calcium (McDougall and Degnan 2018), and only later gained the function of protection from predation (Knoll 2003). One can imagine a similar pattern of emergence involving metazoans and the production of extracellular matrices. When referring to metazoans, one refers not only to the cells of which they are composed but also to the extracellular matrix in which cells live. Indeed, the production of an extracellular matrix has been essential for the transition from unicellularity to multicellularity (Özbek et al. 2010).

This example shows that what is considered internal and external during a transition from unicellularity to multicellularity can shift. Both the environment of a unicellular organism and any material it produces (e.g., the extracellular matrix in a multicellular organism) are external to it. However, some of the very same products are considered part of the multicellular organism. A model of the evolution of multicellularity that does not account for this shift in the boundary between what is external and what is internal must ultimately be incomplete. As such, in a context where many explanations for ETIs have had an internalist bias, taking an external stance permits one to appreciate all the moving parts during such processes. In particular, it allows one to recognize that a boundary shift between what is considered internal and external occurs during the emergence of new levels of individuality.

It should also be noted that the ecological scaffolding perspective does not imply that a scaffold must be abiotic. From the perspective of cells, part of the biotic environment might be regarded as the scaffold that is endogenized. Such transitions typically correspond to those that are egalitarian in nature (Queller 1997), where higher‐level individuality evolves from lower‐level individuals that are phylogenetically distant, as opposed to fraternal transitions, where the higher‐level entity evolves from lower‐level individuals that have a very recent common ancestry. The free‐rider problem, in contrast, has been a primary focus of fraternal transitions. Both experiments and modeling using the ecological scaffolding framework have been concerned with fraternal transitions (but see Doulcier et al. 2020 for an exception). However, an externalist approach to egalitarian transitions may still be of interest as one or more of the partners involved in the transition can be studied as if it were a biotic scaffold for the others. This further highlights the far‐reaching implications of the approach (see also Rainey 2023).

## 5 Conclusion

In this paper, we have presented two contrasting perspectives on ETIs: the prevalent internalist approach and the less common externalist perspective.

Using several examples, we have argued that the emphasis on internal or external factors for explaining ETIs leads to very different perspectives on explanatory factors. In particular, many internalists see the maintenance of multicellular cooperation as the problem most in need of explanation. In contrast, the ecological scaffolding framework instead focuses on the preconditions for the emergence of Darwinian properties.

In light of this, it is evident that there is much to be gained through consideration of both perspectives. Combining insights from both internalism and externalism—and the different questions they raise and solutions that each provide—reinforces the benefits of switching between a purely internalist and a purely externalist approach.

While undoubtedly true, this conclusion underestimates the challenge posed herein. The externalist perspective embodied by the ecological scaffolding framework has been developed specifically to address some perceived shortcomings stemming from internalist explanations. We therefore believe that ecological scaffolding not only provides a novel explanatory approach to the evolutionary puzzle of higher‐level individuality but also already accounts for insights coming from internalist explanations.

## Author Contributions

**Pierrick Bourrat:** conceptualization (lead), funding acquisition (lead), visualization (lead), writing – original draft (lead), writing – review and editing (lead). **Peter Takacs:** conceptualization (supporting), writing – original draft (supporting), writing – review and editing (supporting). **Guilhem Doulcier:** conceptualization (supporting), writing – original draft (supporting), writing – review and editing (supporting). **Matthew C. Nitschke:** conceptualization (supporting), writing – original draft (supporting), writing – review and editing (supporting). **Andrew J. Black:** conceptualization (supporting), writing – original draft (supporting), writing – review and editing (supporting). **Katrin Hammerschmidt:** conceptualization (supporting), writing – original draft (supporting), writing – review and editing (supporting). **Paul B. Rainey:** conceptualization (supporting), writing – original draft (supporting), writing – review and editing (supporting).

## Conflicts of Interest

The authors declare no conflicts of interest.

## Data Availability

The authors have nothing to report.
